# Prevalence and foetomaternal effects of iron deficiency anaemia among pregnant women in Lagos, Nigeria

**DOI:** 10.1371/journal.pone.0227965

**Published:** 2020-01-23

**Authors:** Adegbenga Adetona Ajepe, Kehinde Sharafadeen Okunade, Adebayo Isaiah Sekumade, Ebunoluwa Seun Daramola, Mary Olufunmilayo Beke, Olaolopin Ijasan, Olusola Festus Olowoselu, Bukola Bosede Afolabi

**Affiliations:** 1 Department of Obstetrics & Gynaecology, Lagos University Teaching Hospital, Lagos, Nigeria; 2 Department of Obstetrics & Gynaecology, College of Medicine, University of Lagos, Lagos, Nigeria; 3 Department of Haematology and Blood Transfusion, College of Medicine, University of Lagos, Lagos, Nigeria; University of Dhaka, BANGLADESH

## Abstract

Anaemia in pregnancy is a major health problem and an important cause of adverse foetomaternal outcomes in developing countries. Iron deficiency is the cause of the overwhelming majority of the cases of anaemia in pregnancy. Iron deficiency anaemia (IDA) has been linked with adverse foetal and maternal outcomes. This study investigated the prevalence of IDA and evaluated its effects on foetomaternal outcomes among parturients in Lagos, Nigeria. This was a cross-sectional study that enrolled 220 women aged 15–49 years with singleton gestation at term, between May 1, 2016, and March 31, 2017. Participants were selected by systematic sampling and baseline data were collected through interviews. Venous blood samples were obtained to measure haemoglobin and serum ferritin concentrations, and the associations between IDA (defined as anaemia and iron deficiency) and pregnancy outcomes were examined. A P-value <0.05 was considered as statistically significant. The prevalence of IDA was 12.3%. Routine antenatal iron supplementation (adjusted odds ratio 0.18, 95% confidence interval 0.07–0.46; P = 0.001) and interpregnancy interval of at least 2 years (adjusted odds ratio 0.20, 95% confidence interval 0.05–0.97; P = 0.021) have significant association with IDA. Iron deficiency anaemia was not significantly associated with adverse perinatal outcomes but there were significant associations with increased risk of blood transfusion (P = 0.001) and maternal infectious morbidities such as puerperal pyrexia (P = 0.041) and wound infection (P = 0.020). IDA is still a fairly common condition among parturients in Lagos and it’s mostly associated with maternal peripartum morbidities. Adequate pregnancy spacing through the use of effective contraception and routine antenatal iron supplementations in pregnancy is a recommended preventive measure against IDA and its adverse sequelae. Future studies should adopt the use of transferrin saturation (TSAT) in compliment with serum ferritin assay as a more sensitive marker of iron deficiency.

## Introduction

Anaemia in pregnancy is a major health problem and an important cause of adverse foetomaternal outcomes, especially in developing countries [1,2]. A pregnant woman is considered to be anaemic if her haemoglobin concentration during the first and third trimester of gestation is lower than 11.0g/dL or lower than 10.5g/dL in the second trimester of pregnancy [3]. The reason for these different values, in pregnancy, is that the plasma volume expansion of 40 to 50% exceeds the 20 to 25% increase in red cell mass leading to the physiological haemodilution [4,5]. This physiologic plasma expansion has been linked to favourable pregnancy outcome [6]. However, the value of 10.0g/dL is accepted as the cut-off level in developing countries because adverse foetomaternal outcomes are not usually found above this haemoglobin level in these countries [7]. An estimated 41.8% of pregnant women worldwide are anaemic [1]. The prevalence is even higher on the African continent where an estimated 55.8% of pregnant women are anaemic, and this is attributed mainly to iron deficiency [1]. Other causes of anaemia in pregnancy include nutritional deficiencies such as folate and vitamin B12 deficiencies, as well as chronic medical conditions such as sickle cell disease, chronic kidney disease, chronic liver disease and haematological malignancies. Iron deficiency anaemia (IDA), defined as anaemia accompanied by depleted iron stores and signs of a compromised supply of iron to the tissues [8], occurs across all regions especially in low resource countries and is linked with diminished quality of life, physical and cognitive performance and unfavourable clinical outcomes [9]. It is implicated as the aetiology in more than half of the cases of anaemia in pregnancy.

Iron requirements are greater in pregnancy than in the non-pregnant state as a substantial amount of iron is required in the formation and growth of the placenta, fetus and for the increased red cell mass which places a huge demand on maternal iron stores. Nutritional deficiencies resulting in reduced iron intake, malaria and other parasitic infestations, like hookworm causing chronic blood loss from the gastrointestinal tract, are important causes of iron deficiency in most developing countries [10]. This is further compounded by the fact that many women have varying degrees of iron depletion prior to the onset of pregnancy [3]. In such women, the iron absorption from dietary intake is insufficient to meet the iron requirement in pregnancy and iron deficiency will ensue except in cases in which the pregnant woman takes iron supplementation [3]. There is a stepwise progression towards IDA: initial depletion of iron stores, followed by iron-deficient erythropoiesis (IDE), then a reduction in haemoglobin concentration. Therefore, iron deficiency anaemia represents the end of the spectrum of iron deficiency [11]. Traditionally, serum ferritin is the most widely used marker for evaluation of iron stores as it is not as invasive as bone marrow iron which is the gold standard [12]. In the setting of anaemia, low serum ferritin (<15μg/L) is the most specific laboratory test for iron deficiency [13] and under normal circumstances, it is also a sensitive marker for iron status. However, ferritin is an acute-phase reactant that becomes elevated in response to inflammation just like C-reactive protein (CRP) thus complicating its use for diagnosis in patients with acute inflammatory conditions [14]. Therefore, the standard threshold for iron deficiency does not apply and transferrin saturation (TSAT), a more sensitive marker of iron availability, should also be assessed if possible [14,15]. Our study utilised ferritin as the marker of iron deficiency and excluded women with known or suspected inflammatory conditions from participation.

Iron deficiency anaemia has been linked with increased risk of preterm delivery, postpartum haemorrhage, low birth weight and delayed psychomotor development in infancy [3,9,10,16–18]. However, other studies revealed conflicting conclusions regarding specific outcome measures [18,19]. It is therefore important to evaluate the current prevalence of IDA, its prevailing risk factors and associated foetomaternal outcomes among parturients in Lagos, Nigeria. This will help to inform policies on preventive strategies and optimization of pregnancy outcomes among women in Nigeria.

## Materials and methods

### Study design and setting

This was a cross-sectional descriptive study carried out among parturients at the Labour Ward units of a University Teaching Hospital in Lagos, Nigeria between May 1, 2016, and March 31, 2017. The hospital is a foremost public tertiary health institution that acts mainly as a referral centre for other government-owned and private hospitals in Lagos State. It is on the mainland of Lagos State which has a population of over 9 million inhabitants. The hospital has an established Obstetrics and Gynaecology department which has an annual antenatal clinic attendance and delivery rates of 3000–3500 and about 2200 respectively.

### Study population and recruitment criteria

The participants were recruited using the systematic sampling method in which every third woman who met the inclusion criteria was selected. Inclusion criteria included pregnant women aged 15 to 49 years with a singleton pregnancy who were at a gestational age of at least 37 weeks and gave informed written consent. Pregnant women with diabetes mellitus or gestational diabetes mellitus, preeclampsia, HIV, sickle cell disease, previous and current history of cigarette smoking, multiple pregnancy and polyhydramnios were excluded from the study. Those with infective conditions that may cause a falsely elevated level of serum ferritin such as febrile illness of any cause, recent malaria infection within 1 week of enrolment, abnormal vaginal discharge and prolonged rupture of membranes were also excluded from participation. Further exclusion criteria were conditions that can acutely affect the packed cell volume and haemoglobin concentration such as recent blood transfusion (in the last 2 weeks prior to enrolment) and antepartum haemorrhage.

### Sample size determination and sampling techniques

The sample size (N) was calculated using the formula [20]:

$$N=\frac{Z^{2}P(1-P)}{d^{2}}$$

Using data from a published study by Erhabor et al in Northeast Nigeria [21], the prevalence of IDA (*P*) = 13.5%, the unit normal deviate corresponding to the desired Type I error rate of 5% at 95% confidence interval (*Z*) = 1.96, and a precision (*d*) = 5%. Making provision for a non-response rate of 20%, the minimum sample size required was 215. However, for ease of data collection, collation and analysis, 220 women were enrolled for the study.

### Data collection and laboratory analysis

Prior to the recruitment of eligible participants for this study, written informed consent was obtained after explanation of the nature and purpose of the study, and relevant information such as sociodemographic data, parity, booking status, menstrual history, estimated gestational age, expected date of delivery, and medical history. Other obstetric information such as interpregnancy interval (duration between the last and current pregnancy), self-reported adherence with routine oral iron supplementation and completion of intermittent preventive treatment (IPT) for malaria (defined as the intake of at least two oral doses of Sulphadoxine-pyrimethamine in at least 4 weeks interval after the first trimester) were collected by direct questioning and from the case notes using the proforma designed for the study. The participants’ socioeconomic classes were determined using the women’s educational levels and their partners’ occupations as proposed by Olusanya et al [22]. The woman’s level of education is scored as: tertiary education = 0, secondary level = 1 and primary education or less = 2; while the husband/partner’s occupation is scored as: professional = 1, semi-skilled = 2 and unskilled = 3. The sum of both scores gave the socioeconomic class of the woman. Class 1 represents the highest while Class 5 represents the lowest socioeconomic class with Class 2, 3 and 4 in between.

Following recruitment, two (2) mL of venous blood samples were collected into ethylenediaminetetraacetic acid (EDTA) sample bottle and another 3 mL into a sterile universal bottle from each participant. Serial numbers were assigned to each participant’s proforma and specimen bottles to conceal their identity and ensure confidentiality. The blood samples in the sterile universal bottles were centrifuged to separate the serum from the cellular components. The sera were then well-labelled and the cryovials were stored at a temperature of −20° before final analyses. The EDTA blood sample was used for the estimation of haemoglobin concentration using a Coulter Act 3-part automated Haematology cell counter while the sera separated from the sample in the sterile universal bottles was used for measurement of ferritin levels via the Sandwich enzyme-linked immunosorbent assay (ELISA) technique using the ab108698 –Ferritin Human ELISA kit (Abcam Inc., Cambridge, MA, USA) following manufacturer instructions [23]. Anaemia was defined as haemoglobin concentration <10.0g/dL [7] while Iron deficiency was defined as serum ferritin levels <15μg/L [24]. Women who were found to have iron deficiency anaemia (anaemia and iron deficiency) were offered treatment in collaboration with the Haematologist. At delivery, the infant’s birth status, birth weight and 5-minute APGAR scores were documented. Information on neonatal unit admission and early neonatal deaths were obtained from the neonatal unit. Data on some selected maternal outcomes such as peripartum blood transfusion, wound infection and puerperal pyrexia were also obtained.

### Data analysis

Data obtained was analyzed using SPSS version 23.0 for windows manufactured by IBM, Armonk, NY, USA. Chi-square (*X*) test was used to establish associations in the baseline, risk factors, foetomaternal outcome and maternal outcome while the Fisher’s exact test was used where appropriate. The mean values of normally distributed continuous variables were compared using the independent sample t-test. Logistic regression was done to identify independent risk factors while adjusting for possible confounders. P-value of <0.05 was considered as statistically significant.

### Ethical approval

The study was carried out after obtaining approval from the Health Research and Ethics Committee of the Lagos University Teaching Hospital, Lagos, Nigeria (Approval number–ADM/DCST/HREC/APP/695). Ethical principles according to the Helsinki declaration were considered during the course of the research. Most of the human subjects were adults and those below the age of 18 years were regarded as “emancipated minors” who were legally able to give informed consents by themselves. “Emancipated minor” is a person that is not of legal age to give consent (below 18 years of age in Nigeria) for a research study but who by virtue of marriage, pregnancy, being the mother of a child whether married or not, or has left home and is self-sufficient can be allowed to do so. All the participants read and signed an informed consent form prior to enrolment in the study; the investigators ensured strict confidentiality of all participants’ information. The biological samples were collected and sent for analyses at no cost to the participants and efforts were made to minimize discomfort to the participants during the sample collections and; all participants were given equal attention and optimal care throughout the study and they stand to benefit from the policy that may eventually emanate from the findings of this study. Pregnant women who were diagnosed with iron deficiency anaemia were offered treatment in collaboration with the Haematologist.

## Results

As shown in Table 1, the mean age of participants in the study was 31.5 ± 6.4 years while the mean gestational age at delivery was 37.2 ± 8.8 weeks. A major proportion (70.0%) of the women had up to the secondary level of education while a significant proportion (40.9%) belonged to the upper socioeconomic status (Class 1 and 2). A large proportion of the women (62.7%) were of parity ≥1 with almost 4% being in the grand-multiparous (parity ≥5) category. Most of the women were of the Yoruba ethnic group (36.8%) while the majority (81.2%) were booked antenatal clients of the hospital. Out of the 220 women in the study, 44 (20.0%) were found to be anaemic and of these anaemic women, 61.4% (27/44) had IDA. The overall prevalence of IDA among the study participants was 12.3% (27/220).

**Table 1:** Baseline characteristics of study participants (n = 220)^a^.

Characteristics	Frequency, *n* (%)
**Mean age (in years)**	31.5 ± 6.4
**Mean GA at delivery (in weeks)**	37.2 ± 8.8
**Educational Status**	
No Formal Education	14 (6.4)
Primary Education	52 (23.6)
Secondary Education	99 (45.0)
Tertiary Education	55 (25.0)
**Socioeconomic Status**	
Class 1	37 (16.8)
Class 2	53 (24.1)
Class 3	68 (30.9)
Class 4	37 (16.8)
Class 5	25 (11.4)
**Parity**	
Nulliparous	82 (37.3)
1–4	130 (59.1)
≥5	8 (3.6)
**Ethnic group**	
Yoruba	81 (36.8)
Ibo	65 (29.5)
Hausa	48 (21.8)
Others	26 (11.8)
**Booking Status**	
Booked	180 (81.8)
Unbooked	40 (18.2)
**Haematological Status**	
IDA	27 (12.3)
Non-IDA	17 (7.7)
Non-anaemic	176 (80.0)

Abbreviations: IDA, iron deficiency anaemia; GA, gestational age

^a^ Values are given as mean ± SD or number (percentage) unless indicated otherwise

In Table 2, there was no statistically significant difference between the mean age of women with IDA and those without any form of anaemia (32.5±5.5 vs. 31.9±5.1 years; P = 0.540). The participants’ socioeconomic levels (P = 0.001) and booking status (P = 0.013) were significantly associated with the presence of IDA.

**Table 2:** Sociodemographic characteristics of women with IDA and those without anaemia (n = 203)^a^.

Characteristics	IDA, n = 27	Non-anaemic, n = 176	P-value
**Mean age (in years)**	32.5 ± 5.5	31.9 ± 5.1	0.540
**Mean GA at delivery (in weeks)**	37.4 ± 9.1	38.0 ± 6.2	0.078
**Educational Status**			0.051^b^
No Formal Education	1 (3.7)	13 (7.4)	
Primary Education	12 (44.4)	36 (20.4)	
Secondary Education	10 (37.0)	82 (46.6)	
Tertiary Education	4 (14.8)	45 (25.6)	
**Socioeconomic Status**			0.001^b^
Class 1	2 (7.4)	32 (18.2)	
Class 2	5 (18.5)	43 (24.4)	
Class 3	4 (14.8)	58 (33.0)	
Class 4	7 (25.9)	28 (15.9)	
Class 5	9 (33.3)	15 (8.5)	
**Parity**			0.943
Nulliparous	10 (37.0)	70 (39.8)	
1–4	16 (59.3)	101 (57.4)	
≥5	1 (3.7)	5 (2.8)	
**Ethnic group**			0.115
Yoruba	13 (48.1)	63 (35.8)	
Ibo	7 (25.9)	55 (31.2)	
Hausa	3 (11.1)	41 (23.3)	
Others	4 (14.8)	17 (9.7)	
**Booking Status**			0.013
Booked	18 (66.7)	151 (85.8)	
Unbooked	9 (33.3)	25 (14.2)	
**Total**	**27 (13.3)**	**176 (86.7)**	

Abbreviations: IDA, iron deficiency anaemia; GA, gestational age

^a^ Values are given as mean ± SD or number (percentage) unless indicated otherwise.

^b^ Fisher exact test.

Table 3 shows that iron supplementation during pregnancy (P = 0.001) and the interval between the last and current pregnancy (P = 0.015) were significantly associated with the presence of IDA (P = 0.001). Other factors such as a history of menorrhagia (P = 0.540), use of IPT for malaria in pregnancy (P = 0.806) and duration of breastfeeding in the last pregnancy (P = 0.055) were not significantly associated with IDA.

**Table 3:** Factors associated with iron deficiency anaemia (n = 203)^a^.

Factors	IDA, n = 27	Non-anaemic, n = 176	P-value
**History of menorrhagia**			0.540
Yes	4 (14.8)	19 (10.2)	
No	23 (85.2)	157 (89.2)	
**Iron supplementation**			0.001
Never	9 (33.3)	14 (7.9)	
Rarely	10 (37.0)	22 (12.5)	
Occasionally	5 (18.5)	33 (18.8)	
Fairly	2 (7.4)	41 (23.3)	
Always	1 (3.7)	66 (37.5)	
**IPT for malaria**			0.806^b^
None	6 (22.2)	34 (19.3)	
One dose	1 (3.7)	16 (9.1)	
Two doses	19 (70.4)	121 (68.8)	
≥ Three doses	1 (3.7)	5 (2.8)	
**IPI (in months)**	14.0 ± 3.3	17.0 ± 4.8	0.015
**Breastfeeding duration (in months)**	9.1 ± 2.6	10.8 ± 3.4	0.055

Abbreviations: IDA, iron deficiency anaemia; IPT, intermittent preventive treatment; IPI, interpregnancy interval

^a^ Values are given as mean ± SD or number (percentage) unless indicated otherwise.

^b^ Fisher exact test.

Following a multivariate analysis of the major risk factors of IDA in the study using a binary logistic regression model, iron supplementation in pregnancy (Adjusted odds ratio = 0.18, 95% CI: 0.07–0.46; P = 0.001) and adequate interpregnancy interval (Adjusted odds ratio = 0.20, 95% CI: 0.05–0.97; P = 0.021) were independently associated with about six-fold and five-fold odds of reduction in the prevalence IDA respectively [Table 4].

**Table 4:** Binary logistic regression analysis of the predictive determinants of IDA.

Predictors	Adjusted Odds Ratio	95% CI	p-value
**Socioeconomic class**	1.28	0.74–2.21	0.386
**Iron supplementation**	0.18	0.07–0.46	0.001
**Booking status**	0.95	0.72–5.11	0.960
**Adequate IPI (≥ 2 year)**	0.20	0.05–0.97	0.021

Abbreviations: CI, confidence interval; IPI, interpregnancy interval

As shown in Table 5, IDA was not significantly associated with any adverse perinatal outcome.

**Table 5:** Association between IDA and adverse perinatal outcomes (n = 203).

Perinatal outcomes	IDA, n (%)	Non-anaemic, n (%)	OR (95% CI)	P-value
**Foetal status (n = 203)**				0.568
Dead (n = 8)	2 (7.4)	6 (3.4)	2.26 (0.43–11.86)	
Live (n = 195)	25 (92.6)	170 (96.6)	1.00 (ref)	
**Infant birthweight (n = 203)**				0.724
<2500g (n = 19)	3 (11.1)	16 (9.1)	1.25 (0.34–4.61)	
≥2500g (n = 184)	24 (88.9)	160 (90.9)	1.00 (ref)	
**5-minute APGAR score (n = 195)**				0.619
<8 (n = 10)	2 (8.0)	8 (4.7)	1.69 (0.91–2.82)	
≥8 (n = 185)	23 (92.0)	162 (95.3)	1.00 (ref)	
**Neonatal unit admission (n = 195)**				0.428
Yes (n = 14)	2 (8.0)	12 (7.1)	1.71 (0.45–6.50)	
No (n = 181)	23 (92.0)	158 (92.9)	1.00 (ref)	
**Neonatal death (n = 195)**				0.350
Yes (n = 3)	1 (4.00)	2 (1.2)	3.35 (0.29–38.22)	
No (n = 192)	24 (96.00)	168 (98.8)	1.00 (ref)	

Abbreviations: IDA, iron deficiency anaemia; CI, confidence interval; OR, odds ratio.

However, Table 6 showed statistically significant associations between IDA in pregnancy and increased risks of adverse peripartum outcomes such as blood transfusion (odds ratio = 6.00, 95% CI: 1.90–19.00; P = 0.001), puerperal pyrexia (odds ratio = 5.38, 95% CI: 1.13–25.49; P = 0.041) and wound infection (odds ratio = 5.95, 95% CI: 1.49–23.76; P = 0.020).

**Table 6:** Association between IDA and adverse maternal outcomes (n = 203).

Maternal outcome	IDA, n = 27	Non-anaemic, n = 176	OR (95%CI)	P-value
**Mode of delivery**				0.378
Caesarean	13 (28.2)	69 (39.2)	0.69 (0.31–1.57)	
Vaginal	14 (51.8)	107 (60.8)	1.00 (ref)	
**Blood transfusion**				0.001
Yes	6 (22.2)	8 (4.6)	6.00 (1.90–19.00)	
No	21 (77.8)	168 (95.4)	1.00 (ref)	
**Puerperal pyrexia**				0.041
Yes	3 (11.1)	4 (2.3)	5.38 (1.13–25.49)	
No	24 (88.9)	172 (97.7)	1.00 (ref)	
**Wound infection**				0.020
Yes	4(14.81)	5 (2.8)	5.95 (1.49–23.76)	
No	23(85.19)	171 (97.2)	1.00 (ref)	

Abbreviations: IDA, iron deficiency anaemia; CI, confidence interval; OR, odds ratio.

## Discussion

The prevalence of anaemia and iron deficiency anaemia (IDA) recorded among parturients in this study were 20.0% and 12.3% respectively. The prevalence of anaemia obtained is almost similar to a previous study conducted in the same setting in Lagos [25] but much lower than the WHO reported National estimates of 41.8% [1] and the 35.3% recorded by Anorlu et al [26] where a higher cut-off level of 11.0g/dL was used for the diagnosis of anaemia. The prevalence of IDA recorded in our study is almost similar to the 13.5% prevalence reported by Erhabor et al in a study conducted among antenatal clinic attendees in Sokoto, Northern Nigeria [21]. The contribution of iron deficiency to anaemia in this study (61.4%) is also similar to the finding of 64.0% reported by VanderJagt et al among women in Northern Nigeria [27]. These findings are however much lower than the 90.0% recorded by Bukar et al among newly booked antenatal patients in Gombe, Nigeria [28]. Ferritin has a very low sensitivity in pregnancy and by using a concentration of <15μg/L as the only marker of iron deficiency without a complementary measurement of transferrin saturation (TSAT), we may have missed a huge proportion of the iron-deficient patients and thus the prevalence of IDA obtained from this study (12.3%) may have been a gross underestimation [29].

We reported that iron supplementation was an independent predictor of reduced risk of IDA and this finding was supported by reports from various other studies [13,30] including a systematic review [31]. This is however not unexpected as pregnancy places huge demands on the maternal iron stores coupled with the fact that a significant number of women in the developing countries, where undernutrition is quite prevalent, enter pregnancy with various degrees of iron depletion and deficiencies [3]. Therefore, iron supplementation plays an important role in restoring this depletion in pregnancy. We also noted that a short interpregnancy interval (IPI) less than two year was predictive of IDA in this study and this was thought to be due to the yet to be fully replaced depletion in maternal nutritional stores between one pregnancy and another—effects popularly illustrated by the “maternal depletion syndrome” [32]. This risk of developing anaemia among women with short IPI was also documented in a cross-sectional study conducted by Conde-Agudelo et al in Uruguay [33] but on the contrary, Razzaque et al [34] in a retrospective study conducted in Bangladesh did not find any significant risk of anaemia in pregnancy as a result of a short IPI. However, a systematic review by Conde-Agudelo and colleagues reported no causal relationship between IPI and maternal anaemia [35]. The association between socioeconomic class and IDA as recorded following a bivariate analysis conducted in this study is consistent with the findings from several previous studies [24,26,36,37]. A similar association was also found between the participants’ booking status and the occurrence of IDA and this is consistent with the study by Owolabi et al [38] who reported that unbooked women were twice at risk of developing anaemia in pregnancy compared to booked women. However, these associations may be explained by other independent predictors which are directly or indirectly related to a woman’s socioeconomic and booking status as noted following our multivariate model. For instance, women of higher socioeconomic status are likely to book for antenatal care during their pregnancies and these women will have screening and treatment of anaemia; and routine administration of iron and folic acid which will help to optimize their haematological parameters before term.

IDA in pregnancy has been linked with adverse fetomaternal outcomes [3,9,16]. However, we recorded no statistically significant relationships between IDA and all adverse perinatal outcomes assessed in this study. This is in contrast to the finding by Drukker et al [10] in which IDA was significantly associated with a low 5-minute APGAR score and neonatal unit admission. Aimakhu et al [19] also reported, at variance, a significantly higher number of stillbirths in non-anaemic women when compared to those without anaemia. These inconsistent findings among studies may suggest several complex and multifactorial influences on perinatal outcomes.

In this study, IDA was not statistically associated with the mode of delivery and this correlated with the finding by Aimakhu et al in a study conducted in Ibadan, Southwest Nigeria [19]. These, however, are at variance with the conclusion by Drukker et al in which the IDA was significantly associated with increased risk of Caesarean deliveries [10]. We recorded 6-fold odds of peripartum blood transfusion among participants with IDA compared to the non-anaemic pregnant women and this is consistent with the 5.45 odds of blood transfusion reported by Drukker et al [10]. Generally, anaemic patients tend to tolerate blood loss very poorly and as such may become rapidly unstable haemodynamically with blood loss that would ordinarily not adversely affect a normal patient. IDA has also been linked with increased susceptibility to infection because iron plays an important role in the immunosurveillance of cell-mediated immunity and cytokines function [39] and this was confirmed in our study as we recorded statistically significant associations between maternal infectious complications such as puerperal pyrexia and wound infection.

The current study was hospital-based, limiting the generalizability of the findings to the entire population of pregnant women in Nigeria. Moreover, it was extremely difficult to extract reliable information on the dietary intake of the participants, an important factor that could have had some direct or indirect influence on the associations reported in the study. It is also important to highlight that the associations observed in the study do not necessarily indicate causality. However, this is only one of the few studies among black African women that examined the risk factors and the possible effects of iron deficiency anaemia on pregnancy outcomes. Finally, even though the use of ferritin as a marker of IDA is very specific, it is very insensitive in pregnancy because of the increase in serum C-Reactive Protein (CRP) level, as does the serum ferritin level. Therefore, TSAT should always be ordered as a compliment to improve the sensitivity of serum ferritin assay [29] and we plan to do this in our future studies.

In conclusion, this study showed that the iron-deficiency anaemia in pregnancy is still a fairly common condition and its mostly associated with increased maternal peripartum morbidities. Identifiable risk factors include poor compliance with routine iron supplementation during pregnancy and a short interpregnancy interval. Iron deficiency anaemia also increases the risk of maternal blood transfusion and postpartum infectious morbidity. Therefore, there is a need for effective contraceptive methods to be made available, accessible and affordable to ensure adequate pregnancy spacing in women of reproductive age. Pregnant women should also be encouraged to access antenatal care early to allow for adequate optimization of their haematological status through routine oral iron supplementations and/or parenteral iron therapy prior to labour and delivery in order to obviate the need for blood transfusion and its attendant complications. Finally, future studies should adopt the use of plasma TSAT in compliment with serum ferritin assay as a more objective and sensitive marker of iron deficiency and iron-deficiency anaemia.

## Supporting information

S1 Appendix IRB approval. (DOCX)

S1 Dataset (SAV)
