# Supplementary figures and images for: Prevalence and foetomaternal effects of iron deficiency anaemia among pregnant women in Lagos, Nigeria
Source: PLoS One. 2020 Jan 23;15(1):e0227965. doi: 10.1371/journal.pone.0227965 (PMC6977715; doi:10.1371/journal.pone.0227965)

**
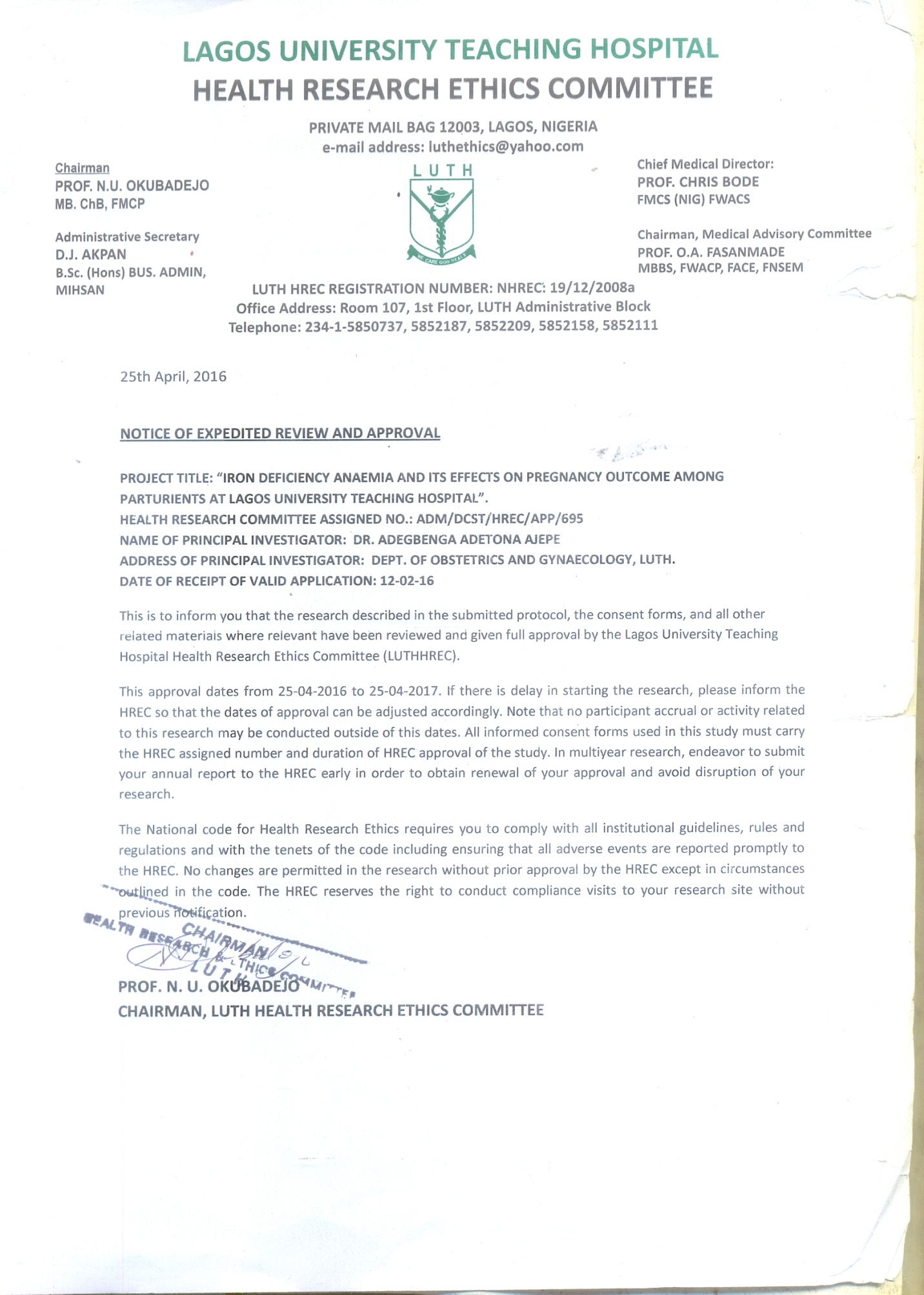
**

Supplement: S1 Appendix — (DOCX) [file pone.0227965.s001.docx]
